# A statistically rigorous sampling design to integrate avian monitoring and management within Bird Conservation Regions

**DOI:** 10.1371/journal.pone.0185924

**Published:** 2017-10-24

**Authors:** David C. Pavlacky, Paul M. Lukacs, Jennifer A. Blakesley, Robert C. Skorkowsky, David S. Klute, Beth A. Hahn, Victoria J. Dreitz, T. Luke George, David J. Hanni

**Affiliations:** 1 Bird Conservancy of the Rockies, Brighton, Colorado, United States of America; 2 Wildlife Biology Program, Department of Ecosystem and Conservation Sciences, W.A. Franke College of Forestry and Conservation, University of Montana, Missoula, Montana, United States of America; 3 Chugach National Forest, Cordova Ranger District, United States Forest Service, Cordova, Alaska, United States of America; 4 Colorado Parks and Wildlife, Denver, Colorado, United States of America; 5 Aldo Leopold Wilderness Research Institute, Missoula, Montana, United States of America; 6 Avian Science Center and Wildlife Biology Program, Department of Ecosystem and Conservation Sciences, W.A. Franke College of Forestry and Conservation, University of Montana, Missoula, Montana, United States of America; Oregon State University, UNITED STATES

## Abstract

Monitoring is an essential component of wildlife management and conservation. However, the usefulness of monitoring data is often undermined by the lack of 1) coordination across organizations and regions, 2) meaningful management and conservation objectives, and 3) rigorous sampling designs. Although many improvements to avian monitoring have been discussed, the recommendations have been slow to emerge in large-scale programs. We introduce the Integrated Monitoring in Bird Conservation Regions (IMBCR) program designed to overcome the above limitations. Our objectives are to outline the development of a statistically defensible sampling design to increase the value of large-scale monitoring data and provide example applications to demonstrate the ability of the design to meet multiple conservation and management objectives. We outline the sampling process for the IMBCR program with a focus on the Badlands and Prairies Bird Conservation Region (BCR 17). We provide two examples for the Brewer’s sparrow (*Spizella breweri*) in BCR 17 demonstrating the ability of the design to 1) determine hierarchical population responses to landscape change and 2) estimate hierarchical habitat relationships to predict the response of the Brewer’s sparrow to conservation efforts at multiple spatial scales. The collaboration across organizations and regions provided economy of scale by leveraging a common data platform over large spatial scales to promote the efficient use of monitoring resources. We designed the IMBCR program to address the information needs and core conservation and management objectives of the participating partner organizations. Although it has been argued that probabilistic sampling designs are not practical for large-scale monitoring, the IMBCR program provides a precedent for implementing a statistically defensible sampling design from local to bioregional scales. We demonstrate that integrating conservation and management objectives with rigorous statistical design and analyses ensures reliable knowledge about bird populations that is relevant and integral to bird conservation at multiple scales.

## Introduction

Over the past two decades, conservation biologists have shifted emphasis from monitoring bird populations at small spatial and temporal scales to considering population responses at eco-regional and decadal scales [1–3]. Monitoring over large spatial extents is necessary for evaluating regional processes of landscape change such as habitat loss, fragmentation, degradation and succession, as well as climate change [1, 4]. Continental programs such as the North American Breeding Bird Survey (BBS; [5, 6]) and Pan-European Common Bird Monitoring Scheme (CBMS; [7, 8]) have contributed much to our understanding of avian population processes operating over large spatial and temporal scales. In addition, adopting a hierarchical frame of reference at local and eco-regional scales is fundamental to interpreting local environmental effects in the context of processes operating at broader temporal and spatial scales [9–11]. For example, monitoring over multiple scales is important for establishing the linkage between small-scale, short-term conservation efforts and large-scale, long-term population trajectories [12, 13]. The cross-scale linkages foster a mechanistic understanding of the interaction between local and regional processes necessary to manage populations [14, 15].

The usefulness of long-term monitoring to large-scale bird conservation depends upon the extent to which monitoring is integrated within a larger framework of management and conservation [2, 10, 16]. Quality monitoring data is essential for conservation science where hypothesis-based predictions are compared to observed population responses to inform management [2, 16]. Statistically defensible monitoring designs for estimating population state variables are needed to address uncertainties for the conservation of bird populations [4, 17, 18]. In addition, monitoring data are essential for the application of adaptive management, with sampling designs integrated within the management and decision making process [4, 19, 20]. The effective conservation and management of bird populations requires a foundation of *a priori* monitoring objectives [2, 10], such as to 1) determine the status and trends of populations at various spatial scales, 2) inform management and policies to achieve conservation, 3) determine causes of population change, 4) evaluate conservation actions, 5) set population objectives and management priorities and 6) inform conservation planning [21].

Although monitoring is an essential component of wildlife management and conservation [1, 22], the lack of regional coordination often undermines its usefulness over large spatial scales [14, 23]. Historically, avian monitoring to address conservation objectives has entailed the creation of a large number of independent monitoring programs at the scale of local management units [10, 24]. However, even when local monitoring programs are co-located within a region of interest, limited spatial representation, inconsistent designs and disparate protocols often preclude reliable inferences about bird populations at larger spatial scales [14, 24, 25]. Limited coordination across disparate monitoring programs, and different organizations and regions results in the inefficient use of monitoring resources and underrepresentation of important regions such as private lands [23].

The lack of rigorous sampling designs is a pervasive problem in many large-scale monitoring programs [2, 25]. Design-based theory for large-scale monitoring programs is well-developed with advantages such as statistically defensible population state variables, minimum estimates of precision and strong inference to regions of interest [26–28]. However, it is often argued that probabilistic sampling is not practical for large scale monitoring [25]. The development of contiguous sampling frames over large regions is often compromised by the incomplete availability of sampling units related to unsafe terrain or landowner permission, and simple random probability sampling is not valid under these frame imperfections [29]. Nevertheless, subjective or convenience samples from atypical areas such as protected reserves or roadways often result in unrepresentative inference and biased population state variables for the region of interest [25, 30]. Sampling solutions are available to address practical difficulties involved with large-scale monitoring designs [29, 31] and these represent preferred alternatives to inferential problems associated with convenience sampling and population indices [25, 27, 32].

Growing concerns about the sustainability of ecosystems, desire from agencies to collaborate across boundaries, and recognition of inherent methodological problems have fostered renewed efforts to improve large-scale monitoring toward a strong science-based foundation for the conservation of birds [2, 21, 23]. In 2008, we designed the Integrated Monitoring in Bird Conservation Regions (IMBCR) Program to overcome many of the historical problems associated with monitoring bird populations at large spatial and temporal scales. The collaboration across organizations and regions focused on leveraging a common data platform over large spatial scales to promote the efficient use of monitoring resources [10, 24]. We designed the IMBCR program using sampling theory to provide a statistical foundation for reliable knowledge about bird populations with the ability to address management and conservation objectives at multiple spatial scales [26–28]. The IMBCR program developed within the context of the U. S. North American Bird Conservation Initiative (NABCI) Monitoring Subcommittee recommendations [21] to 1) integrate monitoring into bird management and conservation, 2) coordinate monitoring programs among organizations and integrate them across spatial scales, 3) increase the value of monitoring information by improving statistical design and 4) maintain bird population monitoring data in modern data management systems.

The IMBCR partnership represents a collaboration between federal and state agencies, Native American Nations, non-governmental organizations and universities working together to develop a strong scientific foundation for achieving bird conservation. The collaboration fits under the umbrella of large-scale conservation programs such as the NABCI [33, 34], Partners in Flight [35], Landscape Conservation Cooperatives [36] and North American Bird Habitat Joint Ventures [37].

Our objectives are to 1) outline the development of a statistically defensible sampling design to increase the value of large-scale monitoring data and 2) provide example applications to demonstrate the ability of the design to meet multiple conservation and management objectives. First, we provide a general outline of the sampling design for the entire IMBCR program from 2010–2015, with a narrowed focus on the Badlands and Prairies Bird Conservation Region (BCR 17) to outline the sampling process within a single BCR. Second, we present two example conservation applications in BCR 17: the first demonstrates hierarchical population estimation to evaluate landscape change for the Brewer’s sparrow (*Spizella breweri*), a species of conservation concern; and the second illustrates multi-scale habitat relationships and distribution modelling to predict the response of the Brewer’s sparrow to conservation practices.

## Materials and methods

### Sampling frame

We defined the target population [28, 38] as all adult individuals of a bird population occupying a BCR [39, 40] during the breeding season for a particular year. We defined the Primary Sampling Unit (PSU) as a 1-km^2^ grid cell and developed the sampling frame for the population by imposing a spatially referenced 1-km × 1-km grid over the BCR [41]. All PSUs in the sampling frame are available to be sampled thus the IMBCR sampling design is able to make valid inference to the entire population in the BCR [28].

**Fig 1:**
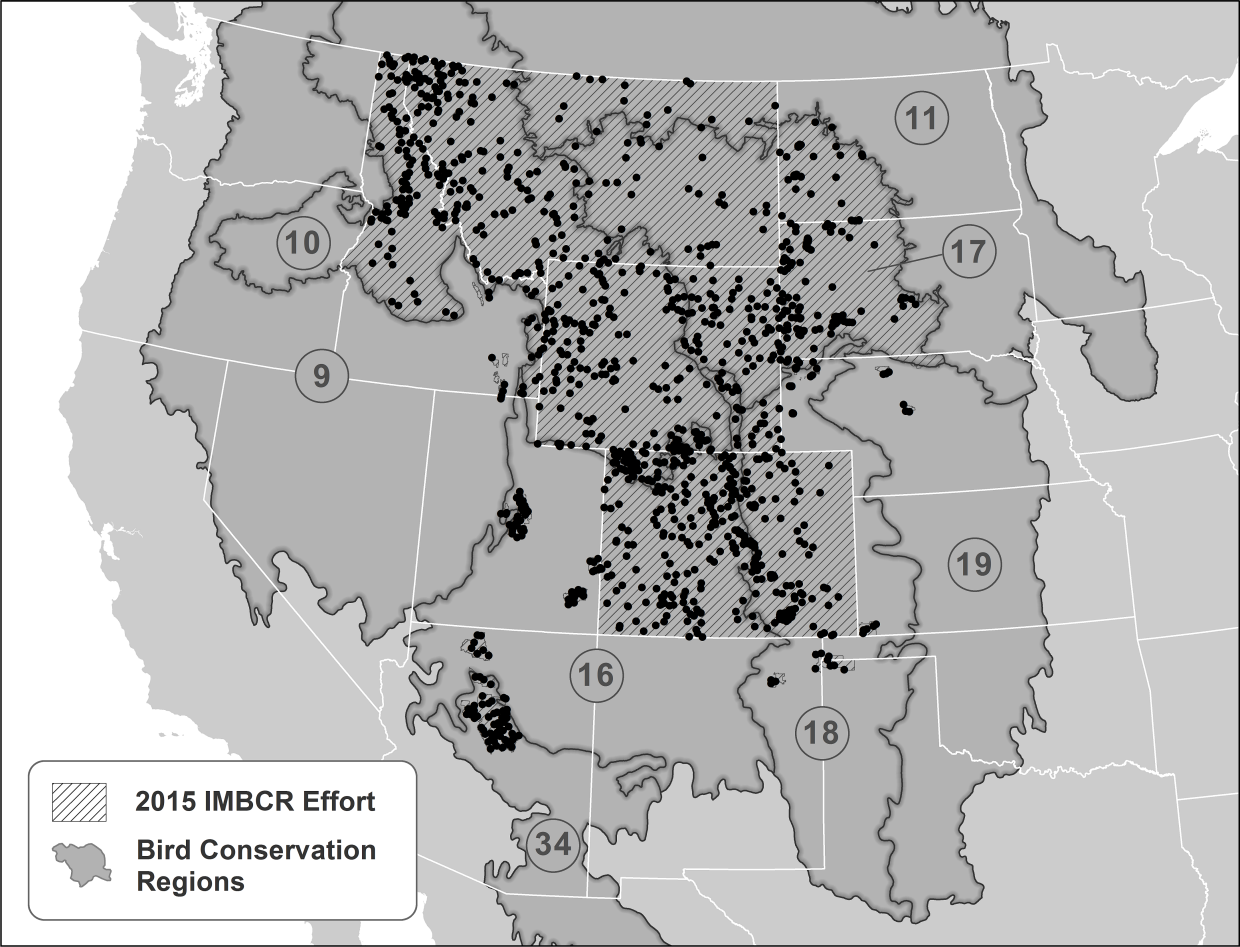
The spatial extent of the 2015 Integrated Monitoring in Bird Conservation Regions Program. The hatched region represents the area of inference and the round symbols represent the locations of Primary Sampling Units (1-km^2^ grid cells) within the 9) Great Basin, 10) Northern Rockies, 11) Prairie Potholes, 16) Southern Rockies, 17) Badlands and Prairies, 18) Shortgrass Prairie, 19) Central Mixed-grass Prairie and 34) Sierra Madre Occidental Bird Conservation Regions.

The 2010 sampling frame of the IMBCR program included Colorado, Wyoming, most of Montana and the entire BCR 17 (1,160,324 km^2^). In 2011, the sampling frame extended to the entire state of Montana (1,165,265 km^2^) with modest changes between 2012 and 2015. The 2015 sampling frame for the IMBCR program encompassed 1,166,973 km^2^, spanning the entire BCR 17 and portions of the Great Basin, Northern Rockies, Prairie Potholes, Southern Rockies/Colorado Plateau, Shortgrass Prairie, Central Mixed-grass Prairie and Sierra Madre Occidental BCRs (Fig 1). The 2015 sampling frame included the states of Colorado, Montana and Wyoming, and portions of Arizona, Idaho, Kansas, Nebraska, New Mexico, North Dakota, Oklahoma, South Dakota, Texas and Utah (Fig 1). We presented the sampling frame for the entire IMBCR program in 2015 as an example of the spatial extent of the Program, with a narrowed focus on BCR 17 to outline the sampling process for a single BCR. We sampled the entire BCR 17 continually between 2010 and 2015, and the area of inference for BCR 17 encompassed 364,497 km^2^ in 2015 (S1 Table).

### Stratification

**Fig 2:**
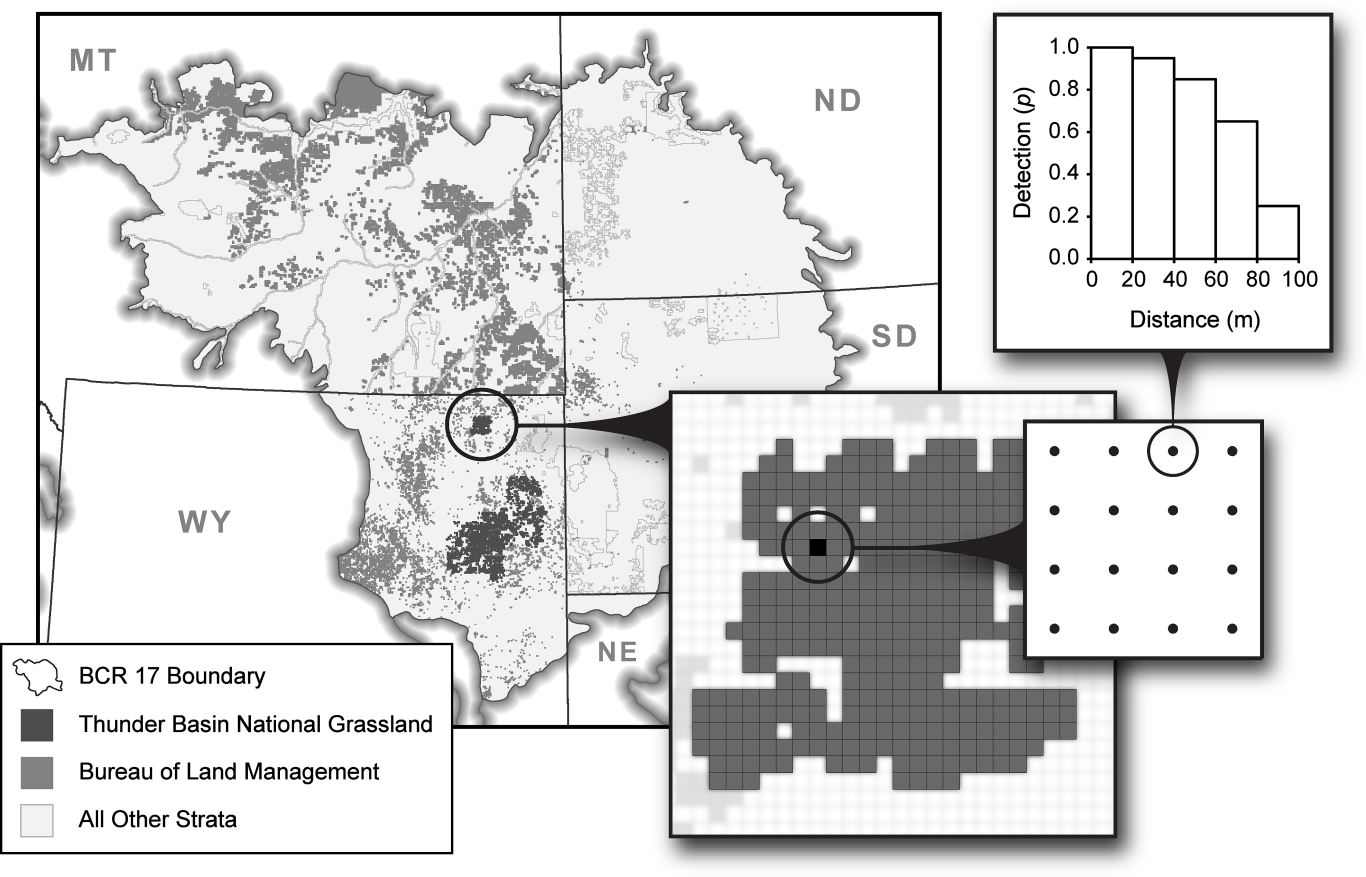
The hierarchical design of the Integrated Monitoring in Bird Conservation Regions Program. The figure illustrates distance intervals nested within Secondary Sampling Units (SSU, point count plots), SSUs nested within Primary Sampling Units (PSU, 1-km^2^ grid cells), PSUs nested within the Thunder Basin National Grassland stratum, and the Thunder Basin National Grassland, Bureau of Land Management and State strata nested within the Badlands and Prairies Bird Conservation Region (BCR 17).

We developed a hierarchical stratification scheme [42] to provide a basis for estimating population state variables at multiple spatial scales (Fig 2). At the first-level, we stratified the sampling frame by the intersection of BCRs and states (e.g., Wyoming portion of BCR 17). At the second-level, we stratified each BCR-by-state region according to the areas of interest, local needs and conservation objectives of the partners. The smallest-order stratification within each BCR-by-state region was based on fixed attributes such as land ownership or management boundaries, latitudinal zones, elevation zones, or major river systems. We included a stratum composed primarily of private land within each BCR-by-state region, which was important for making inference to the entire biological population in the BCR. The flexible stratification scheme allowed each agency or state to stratify their portion of a BCR differently to meet their needs. In addition to providing inference at the level of individual management units, the hierarchical framework allowed strata to be aggregated-up to make inferences at higher-order levels such as BCRs, states and sub-regions, such as agency or private land designations [42].

Here we present year 2015 stratification as an example of the stratification scheme for the IMBCR program, with greater detail for stratification in BCR 17. During 2015 we coordinated with program partners to delineate 171 strata within all or portions of eight BCRs and 13 states (Fig 1). The mean area of the strata for the entire IMBCR program in 2015 was 6,824 km^2^ (SD = 14,367). The stratification scheme for BCR 17 in 2015 included 37 strata (S1 Table), and the mean area of the strata was 9,851 km^2^ (SD = 23,793).

### Sampling design

We developed a two-stage stratified random sampling design [28], in which the first-stage of the design corresponded to the selection of PSUs from the sampling frame of each stratum, and the second-stage represented Secondary Sampling Units (SSU, point count locations) within the selected PSUs. We used a systematic design to locate 16 SSUs within each PSU (Fig 2; [41]). The SSUs were separated by 250 m and located ≥ 125 m from the PSU boundary (Fig 2). The 250 m spacing of the SSUs provides an effective soundscape of 125 m with adequate audible coverage of the PSU for many species. In addition, the 125 m-radius point count plot (4.9 ha) is relevant to the territory sizes of many landbird species and is expected to contain all but the largest territories of most songbird (Passeriformes) species [43, 44]. Because the SSUs within PSUs are dependent by definition [25], we recommend estimation approaches, such as those discussed below, that account for the non-independence of SSUs and avoid double counting of strong singing species. The decision to cluster a relatively large number of SSU subsamples within the selected PSUs was based on increasing sampling efficiency for situations where travel time between widely spaced PSUs is considerable [25]. We require a minimum sample of 4 SSUs per PSU (25%) when private landowners deny permission to survey the SSUs.

Within each stratum, we selected the PSUs using generalized random-tessellation stratification (GRTS; [29]) and thus all PSUs in the sampling frame were ordered such that any set of consecutively numbered units was a spatially balanced sample [29]. We ensured all strata received a minimum of two PSUs to estimate strata-specific variance estimates [28]. We did not specify a minimum sampling fraction for strata because sample allocation was largely determined by partner objectives. Although a minimum sample size of 2 PSUs per stratum was occasionally justified by partner objectives to achieve regional population estimates, inference to an individual stratum requires consideration of the size and variability of a stratum [28, 38], with recommended sample sizes ≥10 PSUs per stratum. Except for cases involving re-stratification, we sampled the same set of PSUs in successive years. However, IMBCR partners can adjust annual sampling intensity within a stratum without compromising the spatially-balanced design, which is particularly useful when budgets or sampling priorities change over time. A clear advantage of GRTS over simple random sampling is the spatially-balanced property of the sample is maintained when access to a PSU is not possible, such as when private landowners deny access permission or dangerous terrain exists [29]. Within the IMBCR sampling design, all areas and vegetation types, including urban and suburban areas, within the sampling frame have a non-zero probability of being included in the sample [28, 45]. Population estimation from a stratified random design requires calculating the probability a sample unit will be selected such that inclusion probabilities are equal for all sampling units in a stratum [38, 46].

Here we present the sampling intensity for year 2015 as an example of sample allocation for the IMBCR program. In year 2015, we sampled 1,225 PSUs in 171 strata throughout the entire IMBCR program. The mean sample size for strata weighted by the number of PSUs in the strata was 9.6 (SD = 7.3), and the mean inclusion probability for the strata weighted by stratum area was 0.0010 (SD = 0.0062). During 2015, we sampled 13,896 SSUs in the second stage of the design throughout the entire IMBCR program, resulting in a mean of 11.3 SSUs per PSU (SD = 2.3). The reasons for incomplete sampling of the SSUs are listed below. During 2015, we sampled 242 PSUs in 37 strata throughout BCR 17 (S1 Table). The mean sample size of PSUs for BCR 17 strata weighted by the number of PSUs in the strata was 9.5 (SD = 7.1), and the mean inclusion probability for the strata weighted by stratum area was 0.0007 (SD = 0.0076). During 2017, we sampled 2,668 SSUs in the second stage of the design throughout BCR 17, resulting in a mean of 11.0 SSUs per PSU (SD = 1.9).

### Data collection protocol

The field observers attended two training programs each year to ensure full understanding of the field protocol, bird and plant identification, and distance estimation using laser rangefinders in a variety of vegetation types. Timing of field visits to the sampling units was structured by latitude and elevation to ensure the visits corresponded to the breeding-season phenology for most species. The observers conducted surveys beginning one half-hour before sunrise and concluding no later than five hours after sunrise. Reasons for observers not surveying all 16 SSUs per grid included running out of time from difficult terrain, wet or windy weather, landowner permission and unsafe terrain. At each SSU, observers conducted six-minute point counts [47]. The observers recorded all non-independent detections of birds (i.e., flocks or pairs of conspecifics in close proximity) as part of a cluster. In addition, observers collected ocular vegetation data within a 50 m-radius of the point count location, including dominant vegetation type, percent cover and mean height of the tree and shrub layers, relative composition of trees and shrubs by species, and height and composition of ground cover types. More detailed information about the data collection protocols can be found at the Rocky Mountain Avian Data Center [48].

Because counts at survey locations do not include all species or individuals present at the sampled locations, we developed data collection protocols at the point locations to accommodate a variety of methods for estimating incomplete detection [45, 49, 50]. The data collection protocol included point-transect distance sampling with distances to independent clusters of individuals measured using a laser rangefinder [49]. The data were recorded separately for each minute interval of the point count, which allowed removal-in-time methods for estimating incomplete detection [45, 50]. In addition, the hierarchical structure of the sampling design (Fig 2) is well suited for estimating detection and availability in hierarchical models of abundance [51], site occupancy [52] and species richness [23]; with distance and minute intervals nested within SSUs, SSUs nested within PSUs, PSUs nested within strata, and strata nested within BCRs (Fig 2).

### Ethics statement

Avian monitoring on U. S. National Park Service (NPS) lands was conducted in accordance with the NPS Institutional Animal Care and Use Committee. Between 2010 and 2015 the Bird Conservancy of the Rockies obtained 170 permits to conduct bird monitoring on 40 parks administered by the NPS, and all permits were archived within NPS Research Permit and Reporting System [53]. Avian monitoring on lands administered by the U. S. Bureau of Land Management, U. S. Department of Defense, U. S. Forest Service, U. S. National Wildlife Refuge System and State Land Trusts did not require permits because of long standing working relationships and agency approval of non-invasive field protocols. Permission to access public or private lands were approved by agency representatives or landowners, respectively and a small number of protected species were detected. The field observers spend < 10 min at SSU point count locations, and the field protocols do not involve animal capture, collections or luring tactics such as call playback, resulting in minimal disturbance to breeding birds.

### Statistical analyses

We estimated annual densities and population sizes for all species with ≥ 80 detections using point-transect distance sampling implemented in the mrds package [49, 54]. We estimated mean population density for PSUs in each stratum (${\hat{d}}_{i}$) by pooling counts among SSUs within PSUs and dividing by effort defined as the number of SSUs within each PSU [25, 55]. For species with ≥ 80 independent detections per year, we fit year-specific, half-normal and hazard-rate detection functions with 10% truncation and no series expansions using Conventional Distance Sampling (CDS). For species with < 80 detections per year, we fit global half-normal and hazard-rate CDS detection functions across years, as well as global detection functions with a factor covariate for year using multiple covariate distance sampling [49]. We evaluated the detection functions for each species using information-theoretic model selection [49, 56]), and used the most parsimonious detection function in the estimation model for each species.

We aggregated estimates of stratum-level densities and population sizes using a weighted one-stage stratified random estimator $\hat{D}=\sum_{i=1}^{n}w_{i}{\hat{d}}_{i}$, where $\hat{D}$ was the aggregated density estimate, *n* was the number of strata, *w*_*i*_ was the proportion of PSUs in stratum *i*, and ${\hat{d}}_{i}$ was the density estimate for stratum *i* [28]. We approximated the variance of the aggregated density and population size estimates using the delta method [57] and Horvitz-Thompson-like estimator [58]. We provided 90% Confidence Intervals (CI) for stratum-level estimates of population density and lognormal CIs for mean density and population size [55].

We estimated site occupancy for all species with >10 detections per year using a multi-scale occupancy model [41, 59] using the RMark [54, 60] interface for program MARK [61]. The parameters of the model included the probability of detection given presence at the SSUs and PSUs (*p*), probability of small-scale occupancy for SSUs given presence at the PSUs (θ), and probability of large-scale occupancy for the PSUs (ψ). We truncated the data at 125 m, which resulted in 16 independent SSUs (4.9-ha point count plots) within each PSU. We pooled the six minute intervals of the point counts into three two-minute time intervals and estimated detection probabilities using a removal design [41, 50]. We aggregated stratum-level estimates of large-scale occupancy using the above, one-stage stratified random estimator, approximated the variance of the aggregated occupancy estimates using the delta method [57] and provided 90% CIs.

#### Example 1: hierarchical population estimation to evaluate landscape change

In the first example, we demonstrate the utility of the hierarchical sampling design for estimating population responses of the Brewer’s sparrow to landscape change within a management unit relative to reference regions in BCR 17. We monitored the population density of the Brewer’s sparrow in the U.S. Forest Service, Thunder Basin National Grassland, WY before and after extensive wildland fires occurred in autumn 2011 (>1,800 ha; [62]). We compared density estimates from the Thunder Basin National Grassland to density estimates in reference regions at multiple scales over time from 2010 to 2015 (Fig 2). The evaluation of a “natural experiment” within a management unit in comparison to reference regions over time allowed the separation of spatial and temporal processes similar to a before-after-control-impact (BACI) design [63]. We predicted a decline in the population density of the Brewer’s sparrow on the Thunder Basin National Grassland following the fires of 2011, but predicted the decline would not be apparent in the reference regions for the Wyoming and BLM portions of BCR 17. In addition, we estimated the population size of Brewer’s sparrows on the Thunder Basin National Grassland, Wyoming portion of the Badlands and Prairies BCR and the entire BCR to evaluate large-scale population consequences from landscape change in the Thunder Basin National Grassland (Fig 2). We estimated effect sizes for the difference between mean population density for 2010 and 2011 before the fires in the autumn of 2011, and mean population density for 2012–2015 following the fires. We estimated the standard errors of the means for the two time periods, and the standard errors of the effect sizes using the delta method [57]. We used 90% CIs with respect to zero to establish magnitude and precision of the effect sizes.

#### Example 2: multi-scale habitat relationships to inform management and conservation

In the second example, we illustrate the applicability of the design for developing habitat and landscape relationships to inform habitat management and landscape conservation in BCR 17. We used presence-absence data at SSUs (4.9-ha point count plots) and PSUs (1-km^2^ grid cells) from 2010 and 2011 to evaluate hypotheses for Brewer’s sparrow occupancy at the local and landscape scales. Multi-scale occupancy is well suited for addressing questions of hierarchical habitat use to understand the primary drivers of a species’ geographic range at the landscape scale, with nested micro-habitat drivers at the local scale [41, 64]. For the purpose of this study, we defined the local scale as processes influencing micro-habitat conditions operating within 4.9-ha SSU plots, and the landscape scale as processes influencing the extent of vegetation operating within 1-km^2^ PSU grid cells [65]. At the local scale, we hypothesized Brewer’s sparrow occupancy of the SSUs would decline with increasing bare ground cover, non-sagebrush shrub cover and woodland canopy cover, and increase with increasing big sagebrush (*Artemisia tridentata*) shrub cover. At the landscape scale, we hypothesized Brewer’s sparrow occupancy of PSUs would increase with the land cover of big sagebrush and mountain big sagebrush (*A*. *tridentata vaseyana*).

At the local scale, we modeled the small-scale occupancy of the SSUs (θ) as a function of covariates for ground, shrub and woodland cover collected at the SSU locations. At the landscape scale, we modeled the large-scale occupancy of PSUs (ψ) as a function of covariates for the land cover of vegetation types within PSUs using the Landfire dataset [66]. The 30 m^2^ grain of Landfire dataset [66] was adequate for evaluating variation in land cover among the 1-km^2^ PSUs, and the 1-km^2^ grain of the resulting species distributions is relevant to all but the smallest management units. We developed *a priori* hypotheses for positive and negative covariate effects on Brewer’s sparrow occupancy [56], but used a data screening step to determine the functional form of the *i* covariates (*x*_*i*_) for small-scale (θ) and large-scale (ψ) occupancy. For each of the occupancy parameters, we forced non-linear [βlog_*e*_(*x*_*i*_)] and quadratic (β*x*_*i*_ + β*x*_*i*_^2^) forms of the covariates into the full model of main effects one at a time, and selected the functional form with the minimum Akaike Information Criterion adjusted for sample size (AIC_*c*_; [56]). We used plausible combinations model selection [67] to identify reduced covariate models for small-scale and large-scale occupancy. In the first step of the plausible combination approach [67], we evaluated all-subsets of the covariates for each parameter, while holding the other parameters constant at the full model. In the first step, we evaluated 6 models for detection probability (*p*), 28 models for small-scale occupancy (θ) and 22 Models for large-scale occupancy (ψ). In the second step of the plausible combination approach [67], we combined all plausible sub-models for the parameters that exhibited a change in AIC_*c*_ (ΔAIC_*c*_) < 2, resulting in a candidate set of 5 models. We created the Brewer’s sparrow distribution map by model averaging [56] the predictions of large-scale occupancy (ψ) from the candidate set of 5 plausible models using the values of the landscape-scale covariates at spatially referenced PSUs (1-km^2^ grid cells) in the sampling frame.

## Results

**Fig 3:**
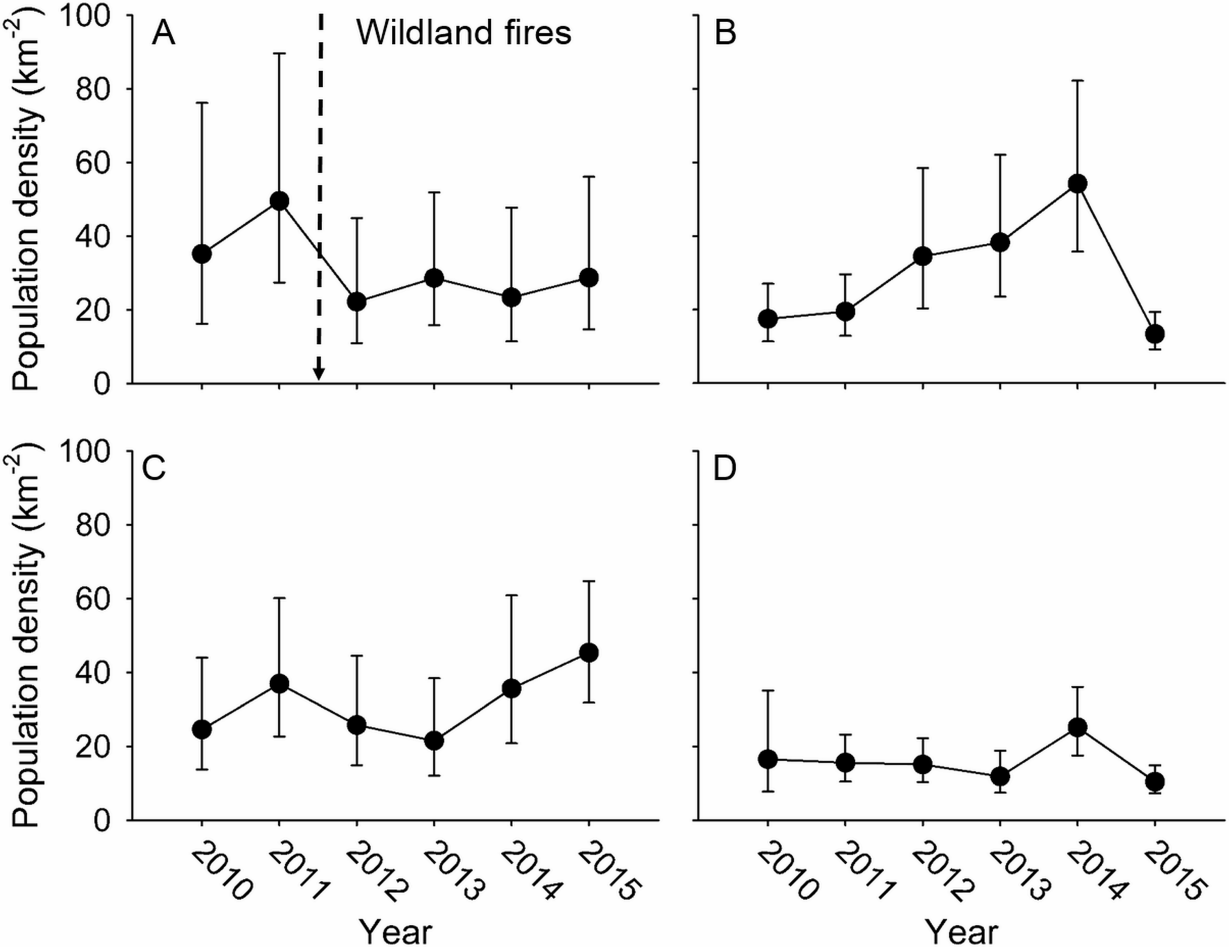
The estimated population density of the Brewer’s sparrow at multiple spatial scales, 2010–2015. (A) U.S. Forest Service, Thunder Basin National Grassland, Wyoming. (B) U.S. Bureau of Land Management lands in the Badlands and Prairies Bird Conservation Region (BCR 17). (C) Wyoming portion of BCR 17. (D) Entire BCR 17. The filled symbols are estimates of population density (km^-2^), the error bars are 90% confidence intervals and the vertical dashed arrow represents the timing of wildland fires in the Thunder Basin National Grassland.

We begin by presenting a summary of density and occupancy estimation for 2015 as an example of annual population estimation from the IMBCR program. During 2015, we detected 306 bird species throughout the entire IMBCR program, and estimated density and population size for 231 species, of which 156 occurred in BCR 17. We estimated large-scale occupancy for 232 species in 2015, of which 154 occurred BCR 17. The Coefficient of Variation (CV) of the density and occupancy estimates provides a diagnostic for the performance of the sampling design. S1 Fig shows the distribution of the CV for 2015 estimates of population density ($\bar{x}=0.596;\;\mathrm{SD}=0.282$) and large-scale occupancy ($\bar{x}=0.522;\;\mathrm{SD}=0.270$) in BCR 17. The hierarchical sampling design (Fig 2) allowed population estimates for the 231 species to be aggregated at various spatial scales as illustrated by the Brewer’s sparrow in BCR 17 (Fig 3). The general location of the bird detections at PSUs can be visualized, and the occupancy and density estimates, as well as the raw counts for SSUs and PSUs, can be retrieved from the Rocky Mountain Avian Data Center [48].

We provided graphs of the most parsimonious detection functions for the Brewer’s sparrow between 2010 and 2015 in S2 Fig as an example of point transect distance sampling to estimate population density from the IMBCR program. Because the Brewer’s sparrow exhibited >80 detections per year, we fit year-specific detection functions for this species. Model selection provided support for hazard-rate detection functions in 2010, 2011, 2014 and 2015, and half-normal detection functions in 2012 and 2013 (S2 Fig).

### Example 1: hierarchical population estimation to evaluate landscape change

In the first example, the mean population density (km^-2^) of the Brewer’s sparrow in 2011 and 2010 on the Thunder Basin National Grassland was 53.8 (SE = 11.7; CI = 37.8, 76.5) before the wildland fires in the autumn of 2011 and mean population density (km^-2^) from 2012 to 2015 was 26.3 (SE = 5.0; CI = 19.0, 35.9) after the fires (Fig 3A). The effect size for the decline in mean population density (km^-2^) after the wildfires was -27.7 (SE = 12.7; CI = -48.3, -6.5). Because the decline in population density (km^-2^) was not observed in the BLM reference (Fig 3B; effect size = 19.6; SE = 7.9; CI = 6.5, 32.7) and Wyoming (Fig 3C; effect size = 1.3; SE = 8.5; CI = -12.7, 15.4) regions in BCR 17 after the autumn of 2011, the BACI design ruled out spatial and temporal variation as a source of the decline, and provided strong evidence the decline was related to landscape change in the Thunder Basin National Grassland. The mean population size of the Brewer’s sparrow in 2010 and 2011 on the Thunder Basin National Grassland before the fires was 243,014 (SE = 52,637; CI = 170,866, 345,624) and the mean population size from 2012 to 2015 after the fires was 117,859 (SE = 22,615; CI = 86,202, 161,141). The effect size for mean population size indicated the Brewer’s sparrow in the Thunder Basin National Grassland declined by 125,155 (SE = 57,290; CI = 61,072, 256,476), a 48% (SE = 14; CI = 30, 78) reduction, after the wildland fires in the autumn of 2011. The population decline of 125,155 amounted to 6% (SE = 3; CI = 3, 15) of the mean population size for the pre-fire years in the Wyoming portion of BCR 17 ($\bar{x}=1,973,128;\mathrm{SE}=460,018$) and 2% (SE = 1; CI = 0, 5) of the mean population size for the pre-fire years in BCR 17 ($\bar{x}=5,905,191;\mathrm{SE}=1,627,157$). Although evaluating population consequences of landscape change over time relative to reference regions is a strength of the IMBCR program, inference to population change must include the possibility that Brewer’s sparrows emigrated from the Thunder Basin National Grassland and redistributed in BCR 17 following the wildfires.

### Example 2: multi-scale habitat relationships to inform management and conservation

**Fig 4:**
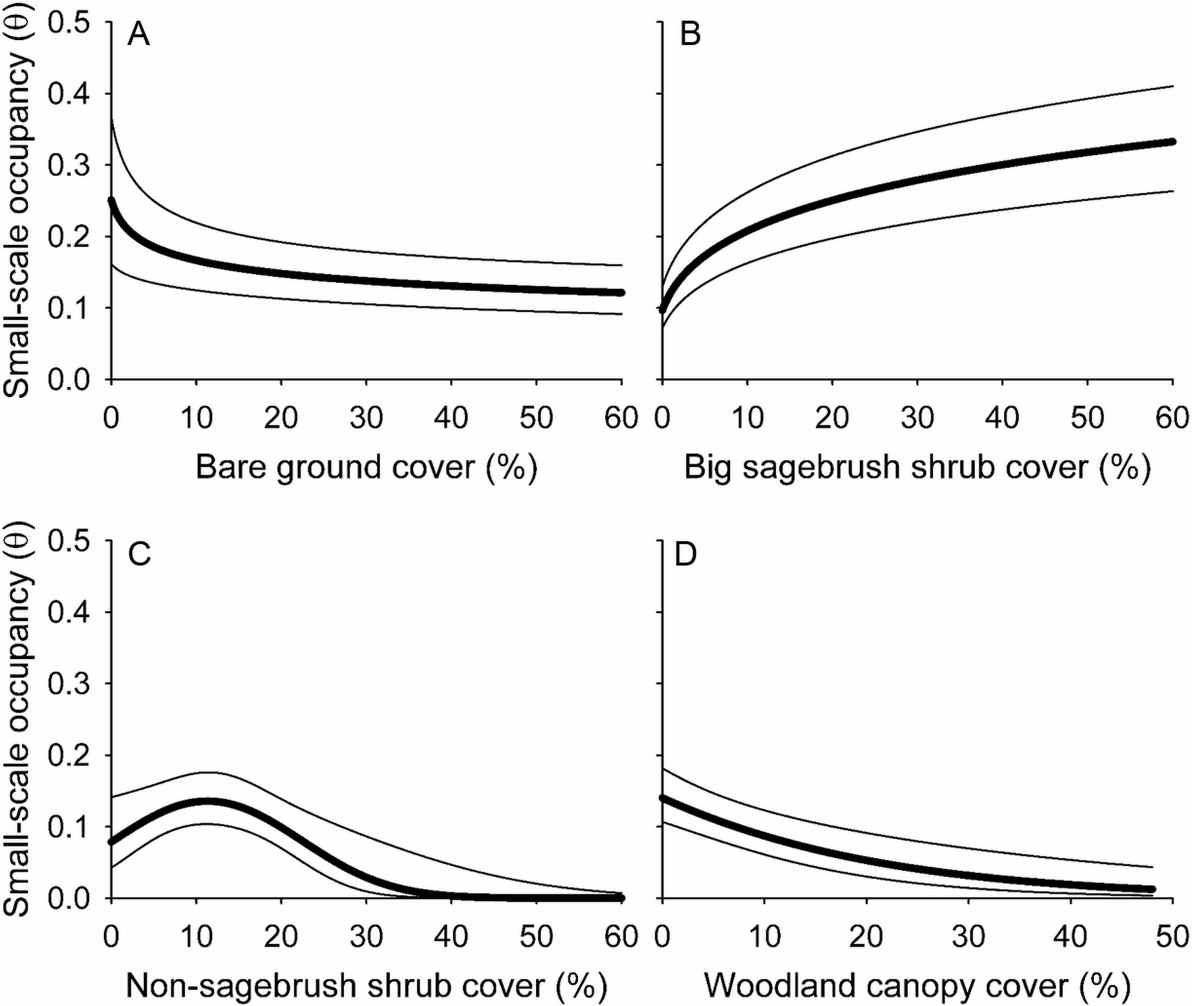
Local habitat relationships for the Brewer’s sparrow in the Badlands and Prairies Bird Conservation Region, 2010–2011. (A) Bare ground cover. (B) Big sagebrush shrub cover. (C) Non-sagebrush shrub cover. (D) Woodland canopy cover. The bold trend lines are model averaged estimates of small-scale occupancy (θ) for Secondary Sampling Units (4.9-ha point count plots) at the mean values of the other covariates in the model and the bounding lines are unconditional 90% confidence intervals.

In the second example, we confirmed the hypotheses that small-scale occupancy of the Brewer’s sparrow at the local scale declined with increasing bare ground (Fig 4A) and woodland (Fig 4D) cover, and increased with increasing big sagebrush shrub cover (Fig 4B; S2 and S3 Tables). The nonlinear effects [βlog_*e*_(*x*_*i*_)] of bare ground (Fig 4A) and big sagebrush shrub (Fig 4B) cover suggested thresholds in the occupancy response (S2 and S3 Tables). We were unable to confirm the hypothesis that the small-scale occupancy of the Brewer’s sparrow declined with increasing non-sagebrush shrub cover (Fig 4C). Conversely, the quadratic effect (β*x* + β*x*^2^) of non-sagebrush shrub cover indicated Brewer’s sparrow occupancy of SSUs increased to an optimum of 11% other shrub cover and declined thereafter (Fig 4C; S2 and S3 Tables). The CIs for the effects of bare ground, big sagebrush shrub cover, quadratic term for other shrub cover and woodland canopy cover excluded zero, indicating measureable effect sizes for these covariates (S3 Table).

**Fig 5:**
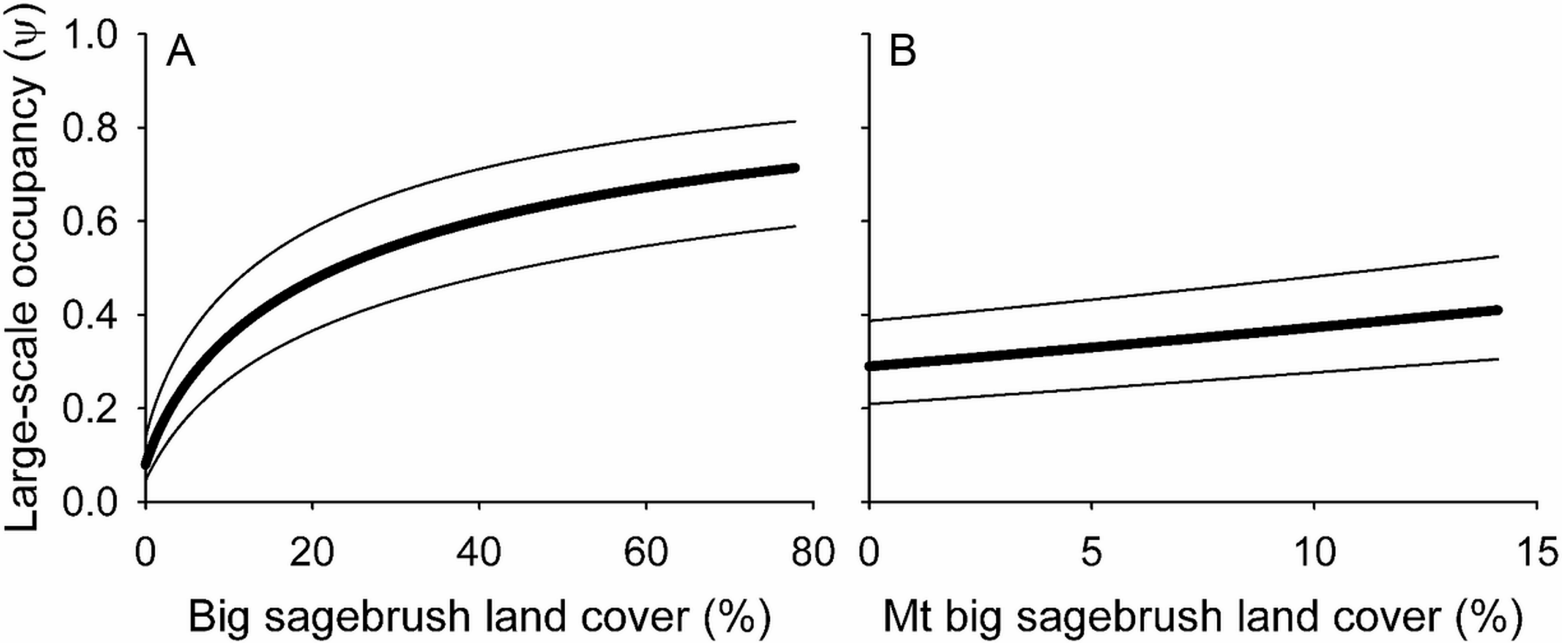
Landscape relationships for the Brewer’s sparrow in the Badlands and Prairies Bird Conservation Region, 2011. (A) Big sagebrush land cover. (B) Mountain big sagebrush land cover. The bold trend lines are model averaged estimates of large-scale occupancy (ψ) for Primary Sampling Units (1-km^2^ grid cells) at the mean values of the other covariates in the model and the bounding lines are unconditional 90% confidence intervals.

**Fig 6:**
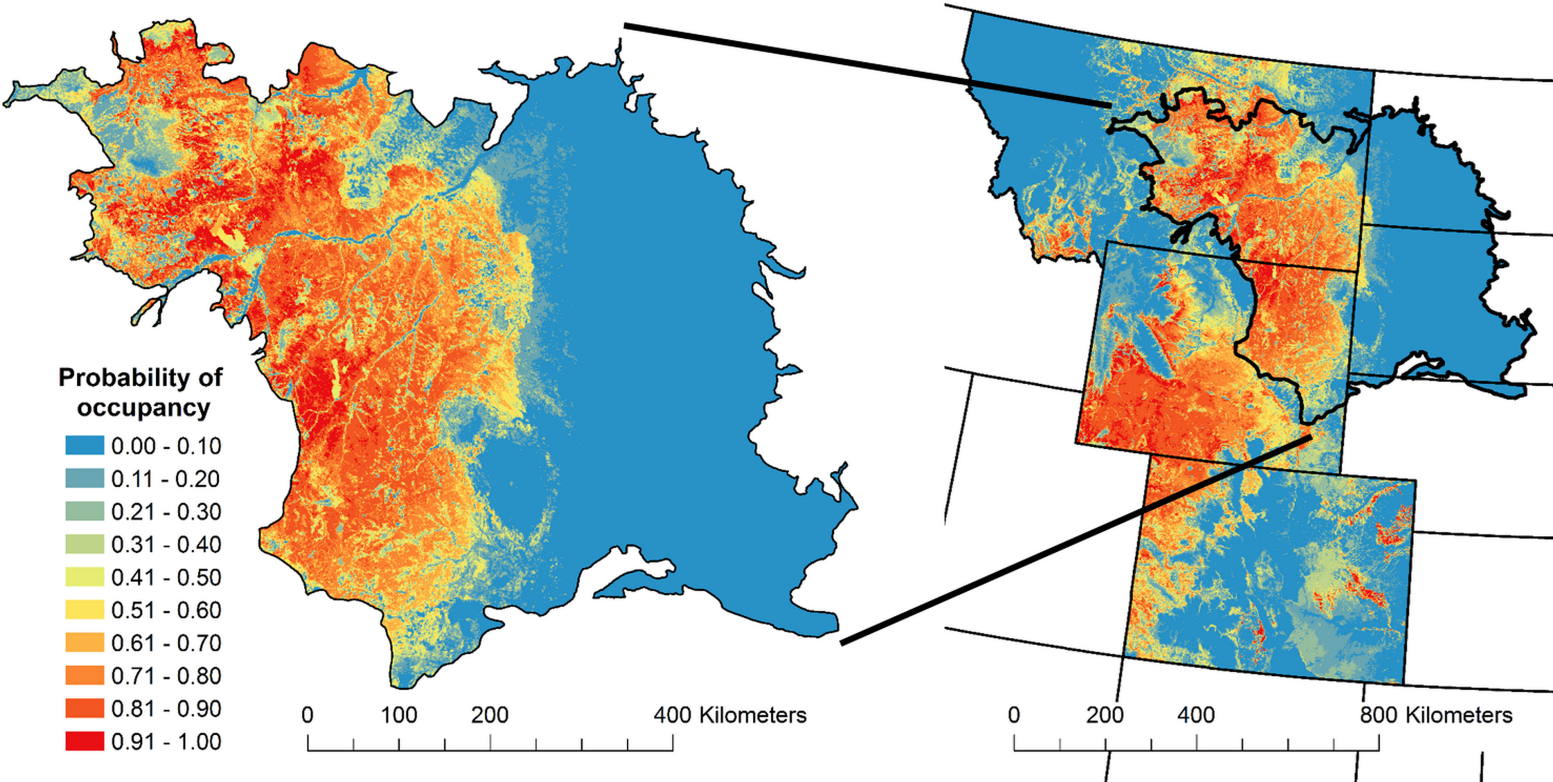
The distribution of the Brewer’s sparrow from the Integrated Monitoring in Bird Conservation Regions Program, 2011. The color ramp for the sampling frame of 1-km^2^ grid cells represent model averaged predictions of large-scale occupancy (ψ) for the most recent distribution in 2011. The inset shows the Brewer’s sparrow occupancy distribution within the Badlands and Prairies Bird Conservation Region at greater resolution.

At the landscape scale, we confirmed the hypotheses that the large-scale occupancy of the Brewer’s sparrow at the PSUs increased with increasing land cover of big sagebrush (Fig 5A) and mountain big sagebrush (Fig 5B; S2 and S3 Tables). The CIs for these effects excluded zero, and the standardized beta parameters provided evidence for a large non-linear [log_*e*_(*x*)] effect of big sagebrush land cover and a smaller linear effect of mountain big sagebrush land cover (S2 and S3 Tables'). The land cover of big sagebrush (Fig 5A) and mountain big sagebrush (Fig 5B) were strong drivers of the predicted occupancy distribution in BCR 17 (Fig 6; S3 Table).

## Discussion

Much has been made of improving the design and analysis of avian monitoring [27, 45, 68], yet the design recommendations have been slow to make their way into large-scale programs [30, 69, 70]. Although it is often assumed that probabilistic sampling is not practical for large-scale monitoring [25], the IMBCR program represents a precedence for implementing a statistically rigorous sampling design over large spatial scales that includes public, tribal and private land ownership [23]. The design of the program uses the principles of sampling theory to identify the target population, sampling frame and sampling unit [28, 46]. In addition, IMBCR employs a probabilistic sampling design, which is important for unbiased population estimates, valid estimates of precision and strong inference to the monitoring region [28]. The GRTS sampling design adjusts the sample selection for frame imperfections and provides a statistically defensible framework for estimating unbiased population state variables [29]. For example, the spatially balanced property of the GRTS sample is maintained when landowners deny permission to access sampling units, and when funding and sampling intensity varies between years [29].

Because avian responses to vegetation, landscape and climate change are scale-dependent [4, 71, 72], it is important to monitor bird populations at multiple spatial scales. The design of the IMBCR program (Fig 2) is well suited for monitoring bird populations at a variety of ecologically relevant scales from home ranges to eco-regions [41] and addressing biological questions within the theory of hierarchical habitat selection [64]. The hierarchical design facilitates the direct comparison of bird populations at local and regional scales, as well as among strata with different levels of human modification [11]. The comparison of population responses at local and eco-regional scales is particularly important for interpreting local environmental effects in context with processes operating at broader temporal and spatial scales [9–11]. For example, population responses at the local scale may not correspond to range-wide population responses for grassland bird species that regularly display nomadic movement in response to spatial weather patterns [73, 74].

The design of the IMBCR program is similar to the UK Breeding Bird Survey [70] and several national schemes within the Pan-European CBMS [8], but departs considerably from the North American BBS [6]. The IMBCR program and several programs in the CBMS [8] feature a sampling frame composed of 1-km^2^ sampling units, which provides sample inclusion probabilities and valid inference to regions of interest. The BBS sampling frame is composed of primary sampling units defined by 1° blocks of latitude and longitude [75, 76]. Because the BBS sampling frame and secondary routes within primary units are restricted to roadways [75, 76], inference about bird populations is limited to roaded areas.

The stratification of the IMBCR sampling frame is based on eco-region boundaries and other fixed attributes, and is useful for evaluating long-term responses of wildlife populations to landscape and climate change [11, 77]. In contrast, the CBMS recommends “habitat” stratification [8], which may not be compatible with the objectives of long-term monitoring [26]. Under stratification by vegetation type, strata boundaries often change with shifting vegetation mosaics and new samples must be drawn, resulting in changing sampling frames and inclusion probabilities over time [26]. Moreover, designs that re-sample shifting strata defined by homogenous vegetation may be incapable of detecting changes in bird populations due to landscape and climate change. The IMBCR design samples vegetation types in proportion to availability can easily accommodate post-stratification [46] by using vegetation data collected at the SSUs and inclusion probabilities for the PSUs. The BBS currently uses stratification by states and ecoregions to allocate sampling effort, but stratification was not a feature of the original design [6, 76].

The IMBCR program and several programs in the CBMS [18] employ data collection protocols that allow direct estimates of population size accounting for incomplete detection and provide valid estimates of precision [27, 45, 49]. Conversely, population estimation from the BBS requires several assumptions about the observation process without valid estimates of precision, and includes the assumption that population size along roads is identical in regions away from roads [25, 30].

Growing concerns about landscape and climate change and uncertainties about the response of bird populations to conservation and management highlight the importance of quality monitoring data over large spatial and temporal scales [1, 4, 11]. We designed the IMBCR program to address the core conservation and management objectives of the monitoring partnership, including federal and state land management agencies, and conservation programs such as NABCI [33, 34], Partners in Flight [35], Landscape Conservation Cooperatives [36] and North American Bird Habitat Joint Ventures [37]. The IMBCR program overcomes the limitations of monitoring in locally disconnected management units by seamlessly coordinating monitoring needs in adjacent strata composed of public, tribal and private landownership [23]. The IMBCR program is consistent with the principles of adaptive monitoring [78] in that the management and conservation objectives can be integrated within a conceptual hierarchical model of how bird populations are structured in space and time [4]. Adopting a hierarchical framework within an eco-regional context is necessary for establishing the linkage between local habitat management and regional bird populations [4, 14].

The example applications illustrate how the IMBCR program can inform the conservation and management of bird populations at multiple spatial scales. The first example showed how patterns of population density at management units can be compared to reference regions to evaluate temporal and spatial response to landscape change in much the same way as a BACI design [63]. The approach shows investment in a rigorous sampling design on the frontend can improve the value of the data to land managers on the backend without requiring complex statistical modelling. The design provides a statistically defensible framework for estimating population size and is able to determine the population consequences of landscape change at multiple spatial scales. The ability to link temporal trends to valid estimates population size at multiple spatial scales is a strength of the program [30]. Because the IMBCR design allows abundance estimation at a variety of spatial scales, we are able to place observed changes in a larger, regional context. For instance, we examined the impact of wildland fires on Brewer’s sparrow abundance over time in the Thunder Basin National Grassland, compared to time series in the Wyoming portion of BCR 17, BLM lands in BCR 17 and entire BCR 17. In the same way, hierarchical population estimation can be used to evaluate the contribution of local management actions to regional bird populations [4]. As bird populations continue to decline, estimates of environmental and anthropogenic impacts on population size are becoming a larger issue in bird conservation [35] and management [4].

The second example showed the IMBCR design is well suited for developing habitat and landscape relationships that can be used to predict species responses to habitat management at local and regional scales [41, 79]. At the local scale, habitat relationships are useful for predicting species responses to habitat management [80, 81]. The results suggest prescribed grazing to reduce bare ground cover (Fig 4A) and tree clearing to reduce woodland canopy cover (Fig 4D) may increase Brewer’s sparrow occupancy, whereas grazing practices that increase bare ground cover (Fig 4A) and shrub management that reduces big sagebrush canopy cover below 20% (Fig 4B) may produce declines in Brewer’s sparrow occupancy. At the landscape scale, predictive species distribution models are often used to prioritize landscapes for conservation action [82], and the results suggest landscapes with greater than 30% land cover of big sagebrush (Fig 5A) have the greatest conservation potential for maintaining high Brewer’s sparrow occupancy (Fig 6). Using the multi-scale approach, the local habitat relationships for the SSUs are nested within the predicted landscape distribution for the PSUs [41]. In this way, the hierarchical design of the IMBCR program provides a framework to go beyond simple landscape prioritization towards conservation planning to prioritize management actions in landscapes [83, 84].

In conclusion, we designed the IMBCR program to 1) integrate monitoring into bird management and conservation, 2) coordinate monitoring programs among organizations and integrate them across spatial scales and 3) increase the value of monitoring information by improving statistical design [21]. The design is well suited for addressing multiple management and conservation objectives at multiple spatial scales [2, 4]. Although adaptive management of land birds has lagged behind shorebirds [85] and waterfowl [86], we anticipate the IMBCR program will play an increasingly important role in the adaptive management [4, 19] and landscape conservation [87, 88] of land bird populations. Coordination across organizations and regions is the continuity that promotes frequent use of essential monitoring data within strong partnerships between policy-makers, land managers, conservationists and scientists [2, 10]. Because the design is based on the fundamentals of sampling theory [28, 38], the program ensures unbiased population estimates, valid estimates of precision and strong inference to bird populations in monitoring region. In addition, the hierarchical design (Fig 2) has a natural connection to hierarchical models [89] and model-based approaches for population and occupancy estimation [17, 18], trend estimation [90], landscape and habitat ecology [1, 80], species distribution modeling [79, 91] and community modeling [23, 92]. Finally, the hierarchical design of the IMBCR program provides a data platform to develop coordinated conservation strategies, prioritize management actions and geographic areas, and effectively address the “what to do” and “where to do it” questions in conservation planning [82].

## Supporting information

S1 Table Strata and sample sizes for the Badlands and Prairies Bird Conservation Region, 2015. The strata, states, strata areas (km^2^), and numbers of Primary Sampling Units (PSU) and Secondary Sampling Units (SSU) in the Badlands and Prairies Bird Conservation Region (BCR 17), 2015. The agency abbreviations are BLM = Bureau of Land Management, USFWS = U. S. Fish and Wildlife Service, USFS = U. S. Forest Service and NPS = National Park Service. The state abbreviations are MT = Montana, ND = North Dakota, ND = North Dakota, NE = Nebraska and WY = Wyoming.
(DOCX)

S2 TableModel selection for multi-scale habitat relationships of the Brewer’s sparrow.Model selection for detection (*p*), small-scale occupancy (θ) and large-scale occupancy (ψ) of the Brewer’s sparrow in the Badlands and Prairies Bird Conservation Region during 2010 and 2011. The model selection metrics are the minimized -2 log-likelihood value [$-{2\log}_{e}(L)$], number of parameters (*K*), Akaike Information Criterion adjusted for sample size (AIC_*c*_), difference between model and minimum AIC_*c*_ value (ΔAIC_*c*_) and AIC_*c*_ weight (*w*_*i*_). Models with ΔAIC_*c*_ < 2 are shown.(DOCX)

S3 Table Parameter estimates for multi-scale habitat relationships of the Brewer’s sparrow. Standardized parameter estimates, standard errors (SE), lower and upper 90% confidence limits (LCL and UCL, respectively) for detection (*p*), small-scale occupancy (θ) and large-scale occupancy (ψ) of the Brewer’s sparrow in the Badlands and Prairies Bird Conservation Region, 2010–2011.
(DOCX)

S1 Fig Coefficient of Variation for density and occupancy in the Badlands and Prairies Bird Conservation Regions, 2015. (A) Population density of 156 species. (B) Site occupancy of 154 species. The bar symbols represent the frequency of species within each class interval 0.138.
(DOCX)

S2 Fig Distance sampling detection functions for the Brewer’s sparrow, 2010–2015. The most parsimonious distance sampling functions for the Brewer’s sparrow from the Integrated Monitoring in Bird Conservation Regions Program, 2010–2015. The vertical bars represent the frequency histograms of detections, and the curves represent year-specific hazard-rate detection functions for 2010, 2011, 2014 and 2015, and year-specific half-normal detection functions for 2012 and 2013.
(DOCX)
