# Supplementary material for: A statistically rigorous sampling design to integrate avian monitoring and management within Bird Conservation Regions
Source: PLoS One. 2017 Oct 24;12(10):e0185924. doi: 10.1371/journal.pone.0185924 (PMC5655431; doi:10.1371/journal.pone.0185924)
Supplement: S1 Table — The strata, states, strata areas (km2), and numbers of Primary Sampling Units (PSU) and Secondary Sampling Units (SSU) in the Badlands and Prairies Bird Conservation Region (BCR 17), 2015. The agency abbreviations are BLM = Bureau of Land Management, USFWS = U. S. Fish and Wildlife Service, USFS = U. S. Forest Service and NPS = National Park Service. The state abbreviations are MT = Montana, ND = North Dakota, ND = North Dakota, NE = Nebraska and WY = Wyoming. (DOCX) [file pone.0185924.s001.docx]

**S1 Table. Strata and sample sizes for the Badlands and Prairies Bird Conservation Region, 2015.**

| Stratum | State | Area | PSU | SSU |
| --- | --- | --- | --- | --- |
| All other lands | MT | 102,779 | 8 | 88 |
| BLM | MT | 25,013 | 4 | 59 |
| USFWS: Charles M. Russell National Wildlife Refuge | MT | 3,709 | 2 | 14 |
| USFS: Custer National Forest | MT | 2,649 | 6 | 68 |
| USFWS: all other lands | MT | 326 | 2 | 31 |
| USFS: Lewis and Clark National Forest | MT | 867 | 3 | 37 |
| Rivers: Missouri; Musselshell; Tongue; Yellowstone | MT | 4,575 | 2 | 18 |
| BLM | ND | 165 | 2 | 26 |
| NPS: Knife River Indian Villages National Historic Site | ND | 5 | 5 | 55 |
| USFS: Little Missouri National Grassland | ND | 4,133 | 5 | 46 |
| All other lands | ND | 48,476 | 4 | 37 |
| USFS: Cedar River National Grassland | ND | 20 | 5 | 55 |
| Select tribal lands | ND | 1,761 | 2 | 19 |
| NPS: Theodore Roosevelt National Park; north unit | ND | 100 | 6 | 45 |
| NPS: Theodore Roosevelt National Park; south unit | ND | 193 | 8 | 88 |
| USFS: Oglala National Grassland | NE | 350 | 4 | 45 |
| All other lands | NE | 1,898 | 3 | 35 |
| USFS: Black Hills National Forest; all other watersheds | SD | 5,009 | 13 | 131 |
| BLM | SD | 831 | 13 | 150 |
| NPS: Badlands National Park; north unit | SD | 428 | 15 | 161 |
| NPS; Badlands National Park; south unit | SD | 539 | 2 | 24 |
| USFS: Buffalo Gap National Grassland | SD | 2,346 | 5 | 53 |
| USFS: Black Hills National Forest; code 7 watersheds | SD | 376 | 3 | 32 |
| NPS: Jewel Cave National Monument | SD | 5 | 5 | 57 |
| NPS: Mount Rushmore National Monument | SD | 6 | 6 | 55 |
| All other lands | SD | 86,853 | 8 | 77 |
| USFS: Fort Pierre National Grassland | SD | 482 | 5 | 62 |
| USFS: Grand River National Grassland | SD | 613 | 5 | 55 |
| Select tribal lands | SD | 5,364 | 2 | 28 |
| USFS: Custer National Forest | SD | 326 | 13 | 153 |
| NPS: Wind Cave National Park | SD | 136 | 14 | 159 |
| All other lands | WY | 52,186 | 26 | 295 |
| USFS: Black Hills National Forest | WY | 1,085 | 3 | 23 |
| BLM: Buffalo Field Office | WY | 2,653 | 7 | 81 |
| BLM: Casper Field Office | WY | 2,695 | 8 | 108 |
| BLM: Newcastle Field Office | WY | 1,025 | 8 | 75 |
| USFS: Thunder Basin National Grassland | WY | 4,520 | 10 | 123 |
| BCR 17 total | 5 | 364,497 | 242 | 2,668 |

The strata, states, strata areas (km^2^), and numbers of Primary Sampling Units (PSU) and Secondary Sampling Units (SSU) in the Badlands and Prairies Bird Conservation Region (BCR 17), 2015. The agency abbreviations are BLM = Bureau of Land Management, USFWS = U. S. Fish and Wildlife Service, USFS = U. S. Forest Service and NPS = National Park Service. The state abbreviations are MT = Montana, ND = North Dakota, ND = North Dakota, NE = Nebraska and WY = Wyoming.
