# Supplementary material for: A statistically rigorous sampling design to integrate avian monitoring and management within Bird Conservation Regions
Source: PLoS One. 2017 Oct 24;12(10):e0185924. doi: 10.1371/journal.pone.0185924 (PMC5655431; doi:10.1371/journal.pone.0185924)
Supplement: S2 Table — Model selection for detection (p), small-scale occupancy (θ) and large-scale occupancy (ψ) of the Brewer’s sparrow in the Badlands and Prairies Bird Conservation Region during 2010 and 2011. The model selection metrics are the minimized -2 log-likelihood value [−2loge(L)], number of parameters (K), Akaike Information Criterion adjusted for sample size (AICc), difference between model and minimum AICc value (ΔAICc) and AICc weight (wi). Models with ΔAICc < 2 are shown. (DOCX) [file pone.0185924.s002.docx]

**S2 Table. Model selection for multi-scale habitat relationships of the Brewer’s sparrow.**

| Model | -2log*_e_*($\mathcal{L}$) | *K* | AIC*_c_* | ΔAIC*_c_* | *w_i_* |
| --- | --- | --- | --- | --- | --- |
| *p*(BCR + year)  θ[log*_e_*(bare) + log*_e_*(big sage) + non-sage shrub + non-sage shrub^2^ + woodland + ^a^]  ψ[BCR + year + log*_e_*(big sage) + mt big sage + ^b^] | 7816.80 | 49 | 7917.87 | 0.00 | 0.296 |
|  |  |  |  |  |  |
| *p*(BCR + year)  θ[year + log*_e_*(bare) + log*_e_*(big sage) + non-sage shrub + non-sage shrub^2^ + woodland + ^a^]  ψ[BCR + year + log*_e_*(big sage) + mt big sage + ^b^] | 7814.96 | 50 | 7918.16 | 0.29 | 0.255 |
|  |  |  |  |  |  |
| *p*(BCR)  θ[year + log*_e_*(bare) + log*_e_*(big sage) + non-sage shrub + non-sage shrub^2^ + woodland + ^a^]  ψ[BCR + year + log*_e_*(big sage) + mt big sage + ^b^] | 7818.50 | 49 | 7919.57 | 1.70 | 0.126 |
|  |  |  |  |  |  |
| *p*(BCR * year)  θ[year + log*_e_*(bare) + log*_e_*(big sage) + non-sage shrub + non-sage shrub^2^ + woodland + ^a^]  ψ[BCR + year + log*_e_*(big sage) + mt big sage + ^b^] | 7807.90 | 54 | 7919.64 | 1.77 | 0.122 |
|  |  |  |  |  |  |
| *p*(BCR * year)  θ[log*_e_*(bare) + log*_e_*(big sage) + non-sage shrub + non-sage shrub^2^ + woodland + ^a^]  ψ[BCR + year + log*_e_*(big sage) + mt big sage + ^b^] | 7810.09 | 53 | 7919.69 | 1.82 | 0.119 |

Model selection for detection (*p*), small-scale occupancy (θ) and large-scale occupancy (ψ) of the Brewer’s sparrow in the Badlands and Prairies Bird Conservation Region during 2010 and 2011. The model selection metrics are the minimized -2 log-likelihood value [$-\text{2log}_{\text{e}}\mathcal{(L)}$], number of parameters (*K*), Akaike Information Criterion adjusted for sample size (AIC*_c_*), difference between model and minimum AIC*_c_* value (ΔAIC*_c_*) and AIC*_c_* weight (*w_i_*). Models with ΔAIC*_c_* < 2 are shown.

^a^ The models for small-scale occupancy included θ[grass cover + log*_e_*(forb cover) + shrub ht + shrub ht^2^ + log*_e_*(forest cover) + vegetation type].

^b^ The models for large-scale occupancy included ψ(grassland + grassland^2^ + sand sage + low sage + salt shrub + elev + elev^2^ + BCR*elev^2^ + latitude + latitude^2^ + BCR*latitude + longitude + longitude^2^ + BCR*longitude).
