# Supplementary material for: A statistically rigorous sampling design to integrate avian monitoring and management within Bird Conservation Regions
Source: PLoS One. 2017 Oct 24;12(10):e0185924. doi: 10.1371/journal.pone.0185924 (PMC5655431; doi:10.1371/journal.pone.0185924)
Supplement: S3 Table — Standardized parameter estimates, standard errors (SE), lower and upper 90% confidence limits (LCL and UCL, respectively) for detection (p), small-scale occupancy (θ) and large-scale occupancy (ψ) of the Brewer’s sparrow in the Badlands and Prairies Bird Conservation Region, 2010–2011. (DOCX) [file pone.0185924.s003.docx]

**S3 Table. Parameter estimates for multi-scale habitat relationships of the Brewer’s sparrow.**

| Parameter | Estimate | SE | LCL | UCL |
| --- | --- | --- | --- | --- |
| *p*(Intercept) | 1.542 | 0.110 | 1.360 | 1.723 |
| *p*(BCR 11) | -0.305 | 0.246 | -0.710 | 0.099 |
| *p*(BCR 16) | -0.298 | 0.181 | -0.597 | 0.001 |
| *p*(BCR 17) | -0.831 | 0.141 | -1.064 | -0.599 |
| *p*(BCR 18) | -1.975 | 0.281 | -2.437 | -1.512 |
| *p*(Year 2011) | -0.255 | 0.118 | -0.450 | -0.061 |
| θ(Intercept) | -1.597 | 0.157 | -1.855 | -1.339 |
| θ[log*_e_*(Bare ground)] | -0.154 | 0.049 | -0.235 | -0.074 |
| θ[log*_e_*(Big sage)] | 0.406 | 0.038 | 0.343 | 0.470 |
| θ(Non-sagebrush shrub) | 0.189 | 0.145 | -0.049 | 0.427 |
| θ(Non-sagebrush shrub^2^) | -0.604 | 0.235 | -0.991 | -0.216 |
| θ(Woodland) | -0.236 | 0.060 | -0.335 | -0.137 |
| ψ(Intercept) | 7.935 | 2.650 | 3.576 | 12.295 |
| ψ(BCR: 16) | 0.209 | 1.294 | -1.920 | 2.338 |
| ψ(BCR: 11 & 17) | 2.977 | 1.659 | 0.248 | 5.706 |
| ψ(BCR: 18) | -1.539 | 3.411 | -7.151 | 4.073 |
| ψ(Year: 2011) | -0.709 | 0.206 | -1.049 | -0.370 |
| ψ[log*_e_*(Big sage)] | 1.169 | 0.137 | 0.942 | 1.395 |
| ψ(Mt big sage) | 0.475 | 0.093 | 0.322 | 0.628 |

Standardized parameter estimates, standard errors (SE), lower and upper 90% confidence limits (LCL and UCL, respectively) for detection (*p*), small-scale occupancy (θ) and large-scale occupancy (ψ) of the Brewer’s sparrow in the Badlands and Prairies Bird Conservation Region, 2010 - 2011.
