# Supplementary material for: A statistically rigorous sampling design to integrate avian monitoring and management within Bird Conservation Regions
Source: PLoS One. 2017 Oct 24;12(10):e0185924. doi: 10.1371/journal.pone.0185924 (PMC5655431; doi:10.1371/journal.pone.0185924)
Supplement: S1 Fig — (A) Population density of 156 species. (B) Site occupancy of 154 species. The bar symbols represent the frequency of species within each class interval 0.138. (DOCX) [file pone.0185924.s004.docx]

A B

**S1 Fig. Coefficient of Variation for density and occupancy in the Badlands and Prairies Bird Conservation Regions, 2015.**

(A) Population density of 156 species. (B) Site occupancy of 154 species. The bar symbols represent the frequency of species within each class interval 0.138.
