# Supplementary material for: A statistically rigorous sampling design to integrate avian monitoring and management within Bird Conservation Regions
Source: PLoS One. 2017 Oct 24;12(10):e0185924. doi: 10.1371/journal.pone.0185924 (PMC5655431; doi:10.1371/journal.pone.0185924)
Supplement: S2 Fig — The most parsimonious distance sampling functions for the Brewer’s sparrow from the Integrated Monitoring in Bird Conservation Regions Program, 2010–2015. The vertical bars represent the frequency histograms of detections, and the curves represent year-specific hazard-rate detection functions for 2010, 2011, 2014 and 2015, and year-specific half-normal detection functions for 2012 and 2013. (DOCX) [file pone.0185924.s005.docx]

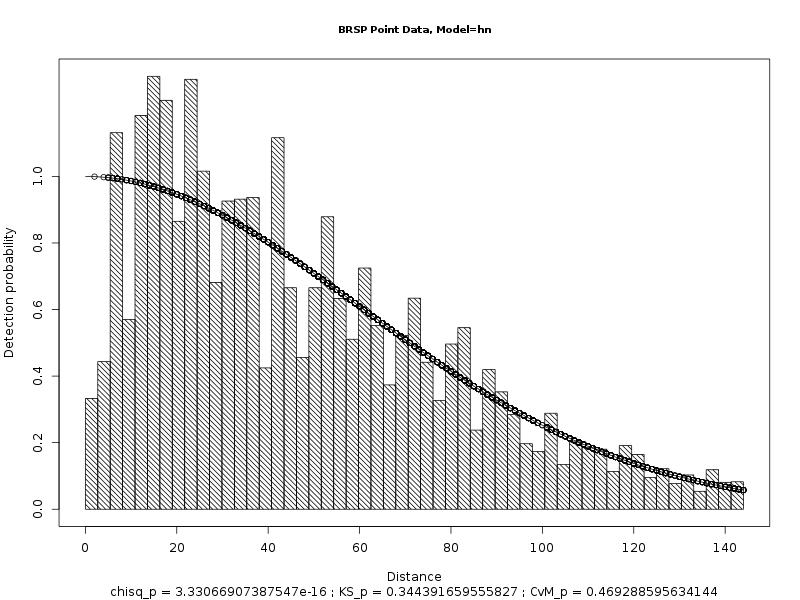

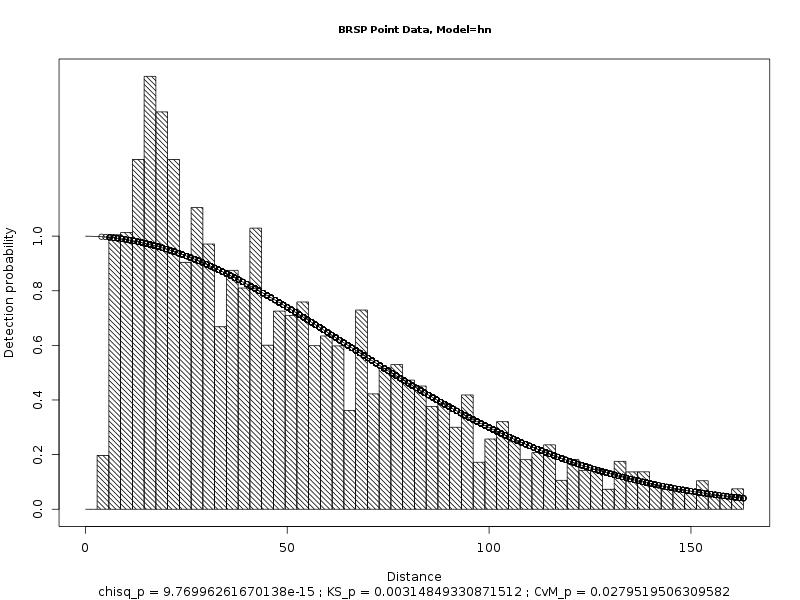

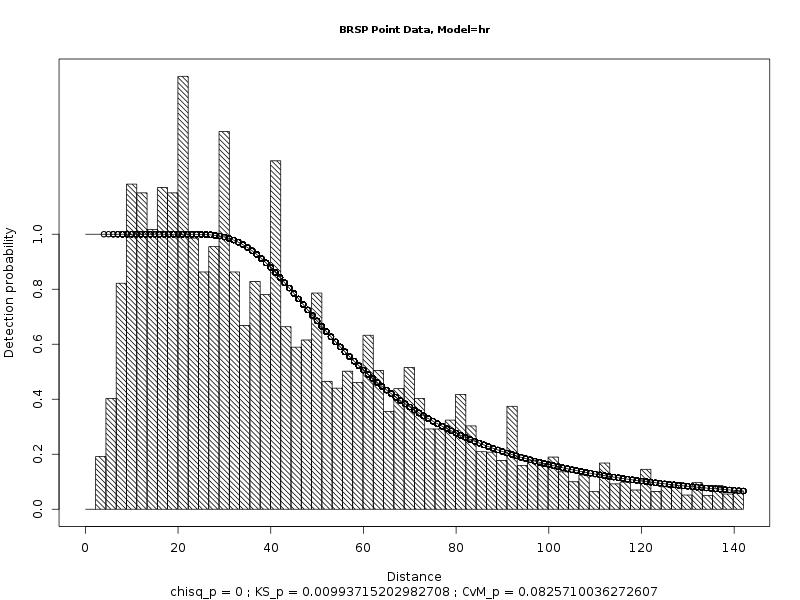

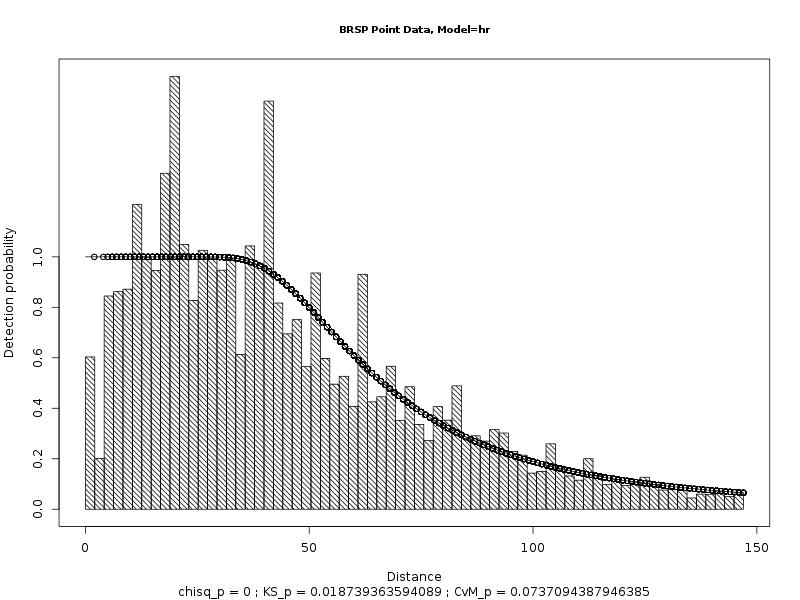

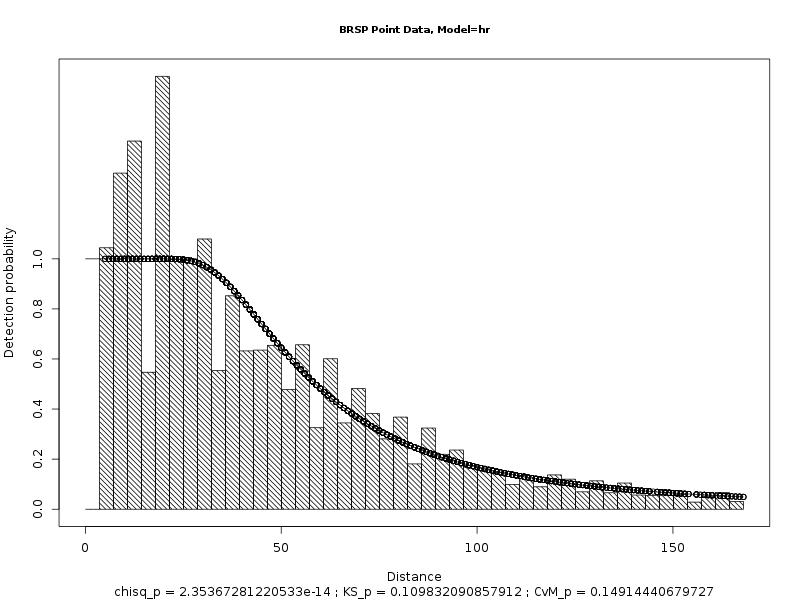

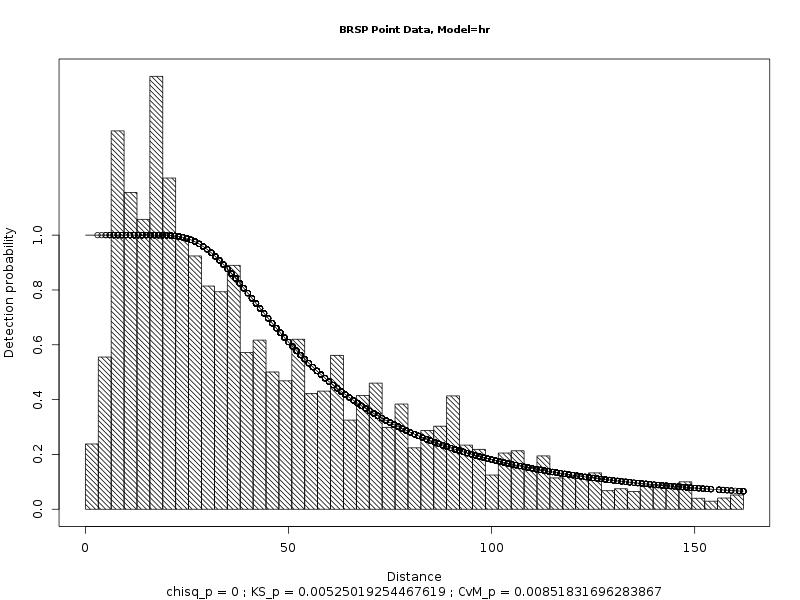


2010 2011

2012 2013

2014 2015

**S2 Fig. Distance sampling detection functions for the Brewer’s sparrow, 2010 - 2015.**

The most parsimonious distance sampling functions for the Brewer’s sparrow from the Integrated Monitoring in Bird Conservation Regions Program, 2010 - 2015. The vertical bars represent the frequency histograms of detections, and the curves represent year-specific hazard-rate detection functions for 2010, 2011, 2014 and 2015, and year-specific half-normal detection functions for 2012 and 2013.
